# Delayed Two-Stage Bentall Procedure: A Safe Technique of Redo Supra-Prosthetic Aortic Root Replacement: A Case Series

**DOI:** 10.3390/jcm14165638

**Published:** 2025-08-09

**Authors:** Maged Makhoul, Nicole Natour, M. Yousuf Salmasi, Jayant S. Jainandunsing, Artur Słomka, Roberto Lorusso, Elham Bidar, Ehsan Natour

**Affiliations:** 1 Cardiovascular Research Institute, Maastricht (CARIM), 6200 Maastricht, The Netherlands; magedmakhoul@gmail.com (M.M.); roberto.lorussobs@gmail.com (R.L.); elham.bidar@mumc.nl (E.B.); 2 Cardiac Surgery Department, Rambam Medical Center, Haifa 3525408, Israel; 3 Radiology Department, University Medical Centre, Herestraat 49, 3000 UZ Leuven, Belgium; nicolenatour@hotmail.de; 4 Department of Surgery, Imperial College, London SW7 2AZ, UK; y.salmasi@imperial.ac.uk; 5 Department of Anesthesiology, University Medical Centre +, 9713 GZ Groningen, The Netherlands; jsjainandunsing@gmail.com; 6 Department of Pathophysiology, Nicolaus Copernicus University in Toruń, Ludwik Rydygier Collegium Medicum, 85-094 Bydgoszcz, Poland; artur.slomka@cm.umk.pl; 7 Department of Hematology and Oncology, National Medical Institute of the Ministry of the Interior and Administration, 02-507 Warsaw, Poland; 8 Department of Cardiothoracic Surgery, Heart and Vascular Centre, Maastricht University Medical Centre +, 6229 HX Maastricht, The Netherlands

**Keywords:** aortic root dilatation, aortic root replacement, redo operation, Bentall procedure

## Abstract

**Background:** Patients presented for complicated redo surgery after previous aortic valve replacement with the indication for aortic root repair due to dilatation or aneurysm. In those cases where the prosthetic aortic valve is in good condition, a valve-sparing procedure might simplify the complicated surgery. The aim of this case series paper is to describe a technique and to show the results of repairing the aortic root without compromising the previously inserted, well-functioning mechanical aortic valve. **Methods:** Between March 2017 and May 2017, 11 patients underwent re-sternotomy with placement on cardiopulmonary bypass with cardiac arrest and exposure of the aortic root. After the aortotomy, the aortic valve was inspected. Subsequently, the aortic sinuses were resected, sparing the coronary ostia buttons. A prosthetic tube was implanted above the preexisting valve. Finally, the coronary ostia were reattached to the tube, turning this procedure into a complete Bentall. **Results:** Echocardiography demonstrated fully functional valves and well-implanted aortic prosthesis. All patients were discharged within ten days post-surgery without any adverse events. **Conclusions:** The delayed two-stage Bentall procedure is a feasible and safe technique that preserves pre-implanted valves and does not cause any distortion of the aortic annulus.

## 1. Introduction

Pathologies of the aortic root have been a significant challenge for cardiologists and cardiac surgeons due to its complex anatomy and function.

Aortic root replacement is usually indicated based on size criteria, as informed by recently updated European and American guidelines [1,2]. Concomitant aortic valve disease, either stenosis or incompetence, is a common consideration during root replacement, first described in 1967 by Bentall and De Bono, with a composite valve graft root replacement. Although less commonly performed, aortic valve-sparing techniques for root replacement were published in the early nineties by David [3] and Yacoub [4] involving aortic valve resuspension or remodeling, respectively. Preserving the native aortic valve, while more technically demanding, avoids the long-term complications of prosthetic valves: namely structural degeneration in biological valves and anticoagulation related complications of mechanical valves.

Patient specificity is key when determining the optimum treatment strategy in aortic valve disease, with or without root pathology. Controversies about whether to replace the mildly dilated aortic root, where AVR is indicated, are present in the literature [5,6,7,8]. Replacing the root (in addition to AVR for hemodynamically significant valve disease) adds to the operative time and thus to the overall risk of open-heart surgery. Therefore, when the root diameter is short of the replacement threshold, many surgeons opt for AVR only and preserve the native root. Despite this, aortic dilatation is becoming a more recognized complication after AVR, affecting 0.6% of all AVR routine procedures [9] and occurring in 10% of cases after BAV replacement [10]. Given the increased risk of type A dissection in these patients with post-AVR root dilatation, redo surgery and root replacement become a necessary procedure. Although not previously well-described, this type of redo aortic root replacement is inevitably higher risk and technically more challenging than an index root replacement.

Due to the complexity of these situations, the accepted treatment is a formal Bentall procedure with explant and replacement of the former prosthetic aortic valve, even if well-functioning, as well as the dilated aortic root using a composite valve graft.

This article aims to show the operative technique and to summarize the results of a new, advantageous, delayed two-stage Bentall procedure where the well-functioning prosthetic aortic valve is maintained, and the dilated aortic root is replaced.

## 2. Materials and Methods

This is a case series paper that describes a redo operative technique and presents the outcome of 11 patients who underwent this procedure: all patients have had one or more cardiac procedures in the past. Since the previous operations took place in different institutes, the operation techniques and the course of hospitalization after these previous operations are incomplete.

### 2.1. Data Collection

Data were collected retrospectively from database units at Groningen Institute, Maastricht Institute (the Netherlands), and Achen Institute (Germany). All cases were performed by a single operator (E.N).

From March 2012 to May 2017, 11 patients were admitted to the hospital due to dilated aortic root or ascending aorta after aortic valve replacement surgery in the past. In all 11 patients, a delayed two-stage Bentall procedure was performed.

The surgical procedure, the rationale for performing this procedure, and detailed outcomes are presented. The follow-up to date was made by medical and nursery personnel.

### 2.2. Definition

The delayed two-stage Bentall procedure was defined as replacing the dilated aortic root with a vascular prosthetic graft and re-implanting the coronary buttons while restoring the well-functioning, previously implanted, mechanical aortic valve.

### 2.3. Pre-Operative Assessment

Choosing suitable patients for a delayed two-stage Bentall procedure is essential since it usually involves high-risk reoperation. Therefore, the decision whether to preserve the previously implanted mechanical aortic valve or not is made by a heart team, including a cardiac surgeon, a cardiologist, an anesthesiologist, and a radiologist.

**Figure 1:**
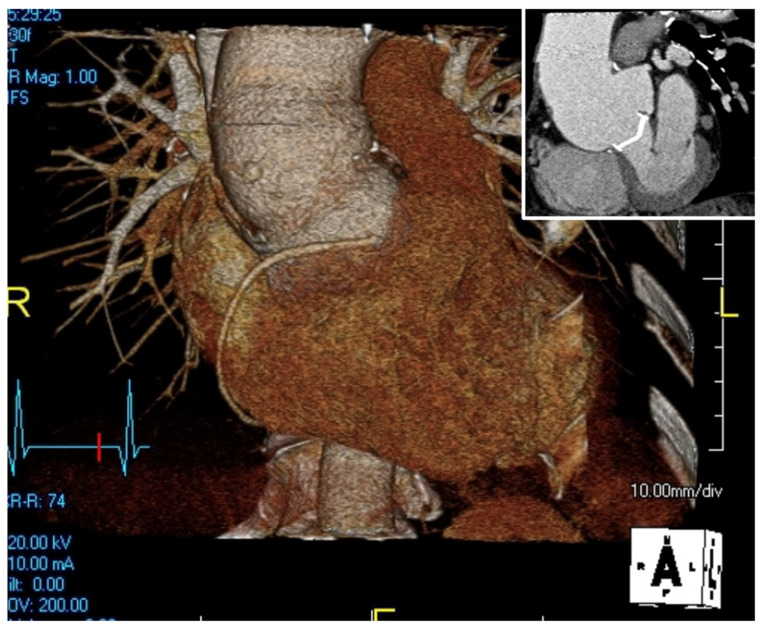
Aortic root aneurysm above the aortic valve prosthesis in 3D reconstruction of the CT scan. The previously implanted mechanical aortic valve can be appreciated in the top corner image—coronal view.

The pre-operative assessment starts with imaging to estimate the severity of the disease, and the structures involved. It starts with echocardiography, including trans-thoracic and trans-esophageal tests, to visualize the aortic valve and assure its well-functionality and hemodynamics, and is followed by a computed tomography angiography (CTA) or a magnetic resonance imaging (MRI) and a 3D image reconstruction to understand the anatomy of the mediastinum, especially in a redo-operation (Figure 1).

The anesthesiologist is then asked to estimate the operative risk, mainly in patients with diminished cardiac function or other high perioperative risk factors.

After vigorous investigations and multi-disciplinary team discussions, the decision whether to preserve the valve or to replace it is made. However, only after opening the aorta and inspecting the valve is the final decision made.

### 2.4. Operative Technique

**Figure 2:**
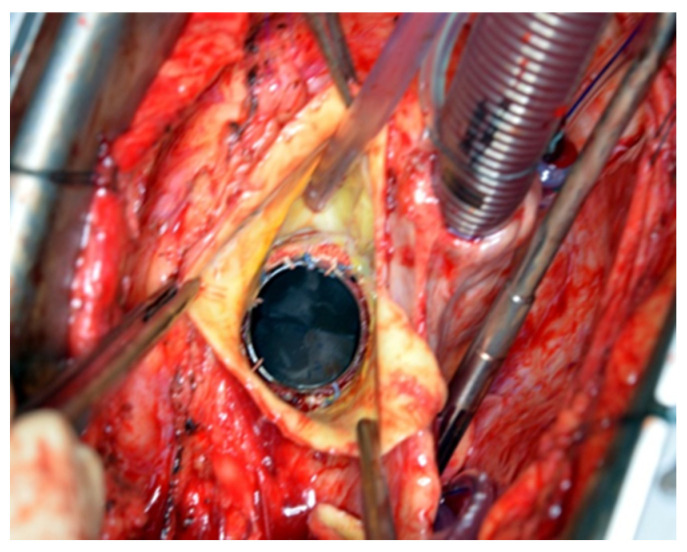
Inspection of the previously implanted mechanical aortic valve prosthesis and coronary ostia. No annular deformity can be seen.

**Figure 3:**
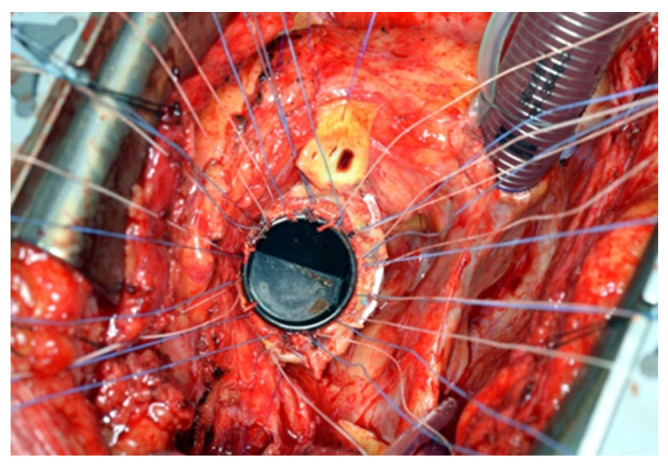
The annulus of the valvular prosthesis is incorporated with the stitches to achieve firmness. Pledged stitches are attached to the previously implanted mechanical aortic valve.

**Figure 4:**
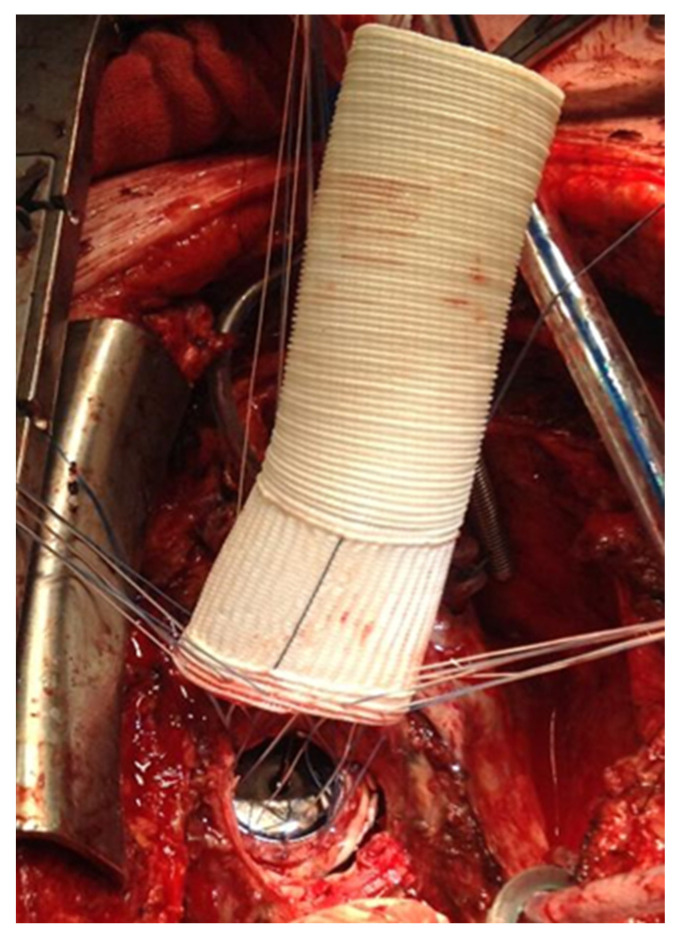
Suture lines are placed through a Dacron tube. The size of the tube is 5 mm larger than the aortic prosthesis annulus.

**Figure 5:**
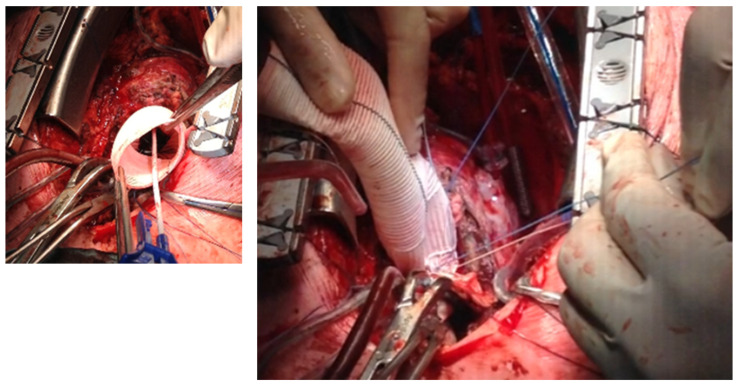
The vascular prosthesis is positioned in the right position (above the prosthesis annulus) and firmly attached to the ring.

**Figure 6:**
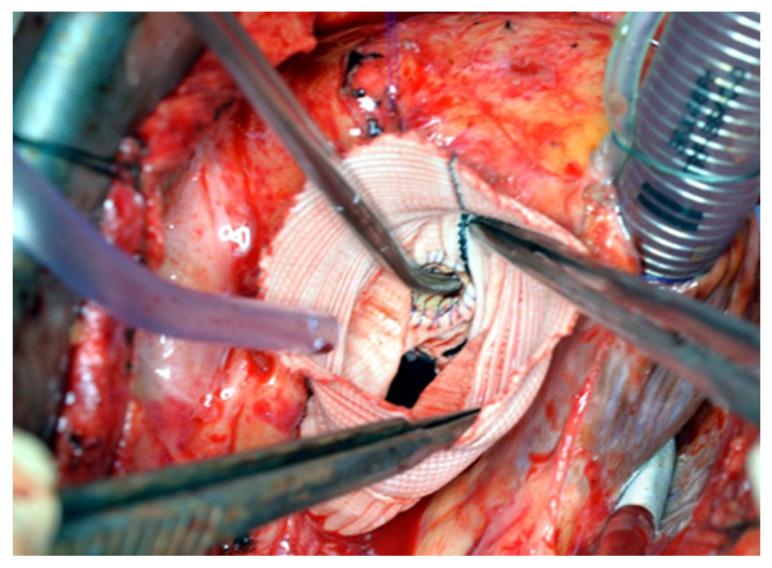
After reimplantation of the coronary ostia, patency is checked.

**Figure 7:**
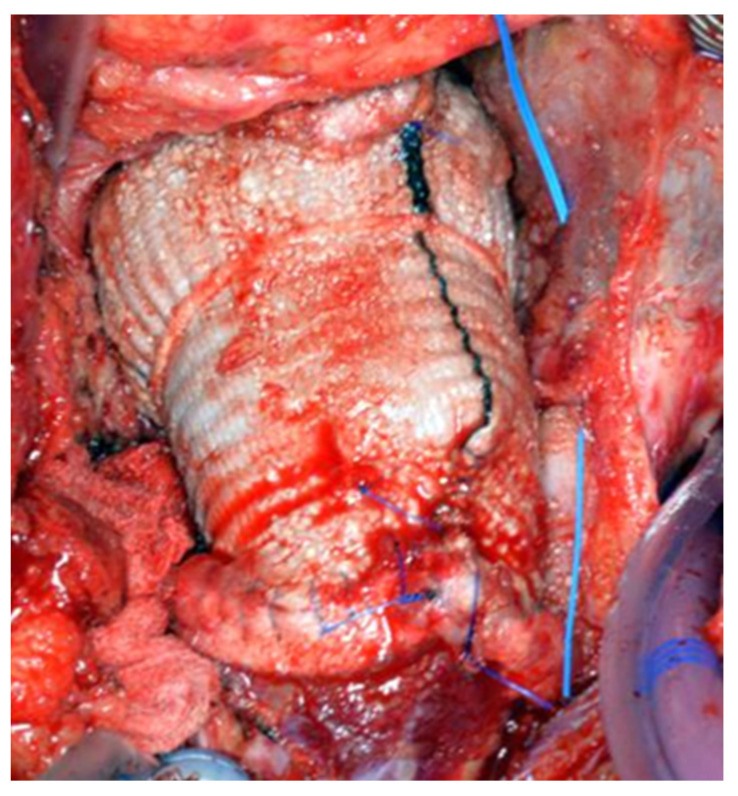
Completion of the delayed two-stage Bentall, after completing the distal anastomosis.

All patients underwent a re-sternotomy approach to access the mediastinum using an oscillating saw to minimize the risk of injuring vital retrosternal structures. The cardio-pulmonary bypass (CPB) machine was centrally connected in all patients via the right atrium to the inferior vena cava, using the two-stage venous cannula (Thin-flex dual stage venous cannulae-Edwards^®^) and via the aorta (EZ glide aortic cannulae-Edwards^®^). Right after cannulation extracorporeal blood flow was achieved, and moderate hypothermia was initiated. An aortic cross-clamp was then applied, and cardioplegia was administered antegradely to the aortic root via an aortic needle (Antegrade cardioplegia cannulae-Edwards^®^) to induce cardiac arrest. The dilated aortic root and ascending aorta were then resected, and the coronary ostia were carefully detached from the aortic root. Direct inspection of the prosthetic aortic valve was performed to rule out any visible pathology (Figure 2). The size of the aortic Dacron graft (Vascutek^®^ Gelweave Valsalva™ Grafts) was selected to be 5 mm larger than the prosthetic aortic valve. Separate 2-0 Ti-Cron^®^ sutures with pledgets were placed to connect the fibrotic aortic annulus and the prosthetic valvular annulus to the aortic graft (Figure 3, Figure 4 and Figure 5). The coronary buttons were then reattached to the graft using 5-0 Prolene^®^, paying particular attention to the geometry of the vessels (Figure 6). A continuous 4-0 Prolene^®^ suture was chosen with pledgets to perform the distal anastomosis (Figure 7).

In two patients (cases 10 and 11), a delayed two-stage Bentall procedure was performed with circulatory arrest to replace the aortic root, ascending aorta, and hemi-arch. Re-sternotomy, cannulation, and aortic resection were performed as previously described. Deep hypothermia was targeted at 25 degrees Celsius. Once hypothermia was achieved, circulatory arrest was started to allow direct inspection of the aortic arch, and cerebral protection was started immediately. After performing the distal anastomosis, the aortic cannula was inserted into the neo-aorta to restart CPB and re-warm the patient. Proximal anastomosis, reconnecting the coronary ostia, de-airing and de-clamping of the aorta, and weaning from CPB was performed routinely.

The Brachiocephalic trunk (BCT) was debranched in two patients (cases 4 and 10). The Dacron aortic graft was distally anastomosed to the aortic arch between the common carotid artery and the BCT, as in hemi-arch replacement, using a 4-0 Prolene^®^ suture with pledgets. A 16 mm vascular graft (Vascutek^®^ Gelweave™ Straight Grafts) was chosen to connect the BCT to the prosthetic ascending aorta. The end-to-side anastomosis was performed using 4-0 Prolene^®^ to connect the vascular tube to the neo-aorta and end-to-end anastomosis, using 4-0 Prolene^®^ with pledgets, to connect the vascular tube to the BCT.

Temporary atrial and ventricular pacemakers were implanted in all patients. Synthetic glue—Coseal^®^—was used prophylactically in all patients to ensure hemostasis. The sternum was closed using steel wires, and the patient was transferred to the cardiothoracic intensive care unit.

## 3. Results

### 3.1. Demographics and Outcome

Altogether, 11 “delayed two-stage Bentall procedure” reoperations took place in three institutes. All patients had open cardiac surgery before this operation, where single or combined cardiac procedures were performed. Among the other procedures, mechanical aortic valves were implanted in all patients, and anticoagulation was prescribed for life. Demographics and cardiac surgical history of the patients are presented in Table 1.

In all cases, the aortic root was severely dilated (53 mm–65 mm). The average time between the first operation and the “delayed two-stage Bentall procedure” was 169 months.

Survival to hospital discharge was 100%, and no major adverse cardiac or cardiovascular events (MACCEs) occurred during the post-operative period.

According to follow-up registries, all patients are in good health. They were all able to answer the phone and report feeling well by themselves at the time of writing this article. The median follow-up time was 61.1 months.

Table 2 summarizes the operative and ICU outcomes of the 11 delayed two-stage Bentall procedure patients.

### 3.2. Two-Stage Bentall Procedure Patients: Pre- and Intra-Operative Considerations

As previously noted, all patients underwent comprehensive pre-operative evaluation, including CTA with 3D reconstruction and echocardiography, to assess chest anatomy before reoperation and evaluate prosthetic valve function. Despite favorable imaging results and confirmed suitability, the final decision to preserve the aortic valve was made intra-operatively after direct inspection of the valve and the annulus.

Preservation criteria included:

(1) Intact valve annulus following dissection,
(2) Proper valve positioning,
(3) Full mobility of the valve leaflets.

In most cases, a two-stage Bentall procedure was performed where the aortic root was replaced while the well-functioning previously implanted mechanical aortic valve was preserved. During surgery, the ascending aorta and aortic root were removed after detaching the coronary ostia. The previously implanted mechanical aortic valve was directly inspected to reassure its adequate function, and a Dacron graft, usually 5 mm larger than the aortic valve diameter, was connected to the prosthetic valve annulus proximally and the distal ascending aorta or as a hemi-ach distally. The coronary ostia were then connected to the aortic graft.

Two cases included BCT debranching, of which one was performed with circulatory arrest. And in one case, the aortic arch needed to be replaced together with the ascending aorta and the root. This was also performed with circulatory arrest (Table 2).

## 4. Discussion

Aortic root dilatation is a known entity after AVR. Many authors describe surgical techniques to replace the dilated aortic root with the prosthetic aortic valve, including the most commonly used, the Bentall procedure [11]. Other authors published their experiences as a single case report or an idea describing the completion of the Bentall procedure and valve-sparing aortic root replacement after AVR [12,13]. However, the delayed two-stage Bentall technique described in this article was never published before in a series of patients.

While aortic root replacement techniques are well-established in cardiac surgery, many smaller centers or younger surgeons avoid performing them. On the one hand, the Bentall procedure and valve-sparing root replacement procedures are surgically demanding; on the other hand, they are life-saving procedures. Nevertheless, in redo settings it is more complicated. Here comes the main advantage of the two-stage Bentall procedure.

Careful patient selection is essential before performing the delayed two-stage Bentall procedure; the prosthetic aortic valve patency is a significant concern. The final decision on whether to proceed with this technique or not can only be made after the surgeon directly inspects the prosthetic aortic valve based on the echocardiographic results previously performed.

Mortality rates after root replacement techniques are low, as are significant complications like bleeding and stroke [14,15]. These data are comparable with the data published in our experience, where the post-operative mortality rate up to 4 years follow-up was 0 and the major post-operative complication rate was 0. Nevertheless, we acknowledge that this case series is limited by the small sample size, the retrospective design, and the absence of control group. For that, further studies are needed to confirm these findings.

Preserving a well-functioning previously implanted prosthetic aortic valve during a delayed two-stage Bentall procedure has several advantages when compared to other aortic root replacement surgeries after a previous mechanical AVR: avoidance of further trauma to the already distressed and fibrosed aortic annulus from previous surgery means a lower chance of inducing atrio-ventricular block. Furthermore, attaching the prosthetic aortic graft to the previously implanted prosthetic mechanical valve is more likely to be a stronger connection than attaching it to the patient’s native tissue. Since less surgery is required in the aortic annulus during the two-stage Bentall technique, there is expected to be less post-operative bleeding. It is worth mentioning that even subvalvular pannus formation could be used as reinforcement tissue with this technique.

Considering the operative timing, preserving the valvular prosthesis would inevitably reduce CPB time and aortic cross-clamp time, yet another important advantage for the patient overall. In fact, according to published papers on Bentall procedure experiences [16,17,18,19], the mean CPB time varied between 161.2 ± 83 and 246.9 ± 89.8 min, and the mean aortic cross-clamp (ACC) time varied between 115 ± 60.4 and 170.3 ± 63 min. According to our experience, the mean CPB time was 200.1 ± 19.7 min, and the mean ACC time was 153.8 ± 17.2 min.

## 5. Conclusions

A delayed two-stage Bentall operation is a safe and feasible procedure. It could replace the formal root replacement techniques when the aortic valve prosthesis is in good condition.

## Figures and Tables

**Table 1:** Patient’s demographic details.

Patient Nr.	Institute	Age	Gender	Cardiac Surgical History (Year)	Aortic Root Diameter (Mm)	Date of Surgery
1	Groningen	56	F	MVP + AVP (1995) AVR + ascending aortic replacement (1997)	65	03/2012
2	Groningen	52	F	AVR (1994) AVR + MVP (1999) pacemaker implantation (2006)	58	12/2015
3	Groningen	54	M	AVR + MVR (2001)	55	06/2014
4	Groningen	47	M	AVR + MVP (1998)	55	09/2014
5	Groningen	46	M	AVR (2003) pacemaker (2008)	59	01/2015
6	Groningen	55	M	AVR (1998)	53	09/2014
7	Groningen	63	M	AVR (2007)	55	11/2013
8	Achen	64	M	AVR + MVR (1997) AVR (2007)	62	01/2017
9	Achen	58	M	AVR (2006)	61	03/2017
10	Maastricht	53	M	AVR (2003)	75	06/2016
11	Achen	57	M	AVR (2004)	67	05/2017

MVP—mitral valve plasty, AVP—aortic valve plasty, AVR—aortic valve replacement, MVR—mitral valve replacement.

**Table 2:** Patient outcomes.

Patient Nr.	Procedure	CPB Time (Minutes)	ACC Time (Minutes)	Circulatory Arrest (Minutes)	Ejection Fraction (%)		Peri-Operative RBCs (Units)	Days to Hospital Discharge
					Pre-Operative	Post- Operative		
1	Re-redo delayed two-stage Bentall.	233	183	No	45–50	50	2	8
2	Re-redo delayed two-stage Bentall	205	165	No	45–60	45–60	0	16
3	Redo delayed two-stage Bentall	193	144	No	50–60	50–60	0	10
4	Redo delayed two-stage Bentall + BCT * de-branching	313	169	No	55–60	55–60	0	5
5	Redo delayed two-stage Bentall	201	162	No	40–45	50	0	6
6	Redo delayed two-stage Bentall	172	137	No	55–60	55	0	8
7	Redo delayed two-stage Bentall	197	151	No	55–60	55–60	0	5
8	Re-redo Delayed two-stage Bentall	195	135	No	70	70	2	8
9	Redo delayed two-stage Bentall	180	130	No	60	60	0	10
10	Redo delayed two-stage Bentall + BCT de-branching	308	174	Yes (47)	60–65	60–65	23	10
11	Redo delayed two-stage Bentall + arch replacement	285	190	Yes (52)	70	70	3	11
	Mean ± SD	217.4 ± 45.4	157.1 ± 20.9					

* BCT—brachiocephalic trunk, CPB—cardio-pulmonary bypass, ACC—aortic cross-clamp, RBCs—red blood cells.

## Data Availability

The original contributions presented in this study are included in the article. Further inquiries can be directed to the corresponding author(s).

## Abbreviations

The following abbreviations are used in this manuscript:

AVR	Aortic valve replacement
CTA	Computed tomography angiography
MRI	Magnetic resonance imaging
CPB	Cardio-pulmonary bypass
BCT	Brachio-cephalic branch
MVP	Mitral valve prolapse
AVP	Aortic valve prolapse
MVR	Mitral valve replacement
MACCE	Major cardiac and cardiovascular event
ICU	Intensive care unit
ACC	Aortic cross clamp
RBCs	Red blood cells
